# Investigating the Impact of Social Media on Body Image and Eating Disorders Among Individuals in Arab Countries: A Literature Review

**DOI:** 10.7759/cureus.95309

**Published:** 2025-10-24

**Authors:** Basmalah Naji, Mohamed Naji, Salim Fredericks

**Affiliations:** 1 School of Medicine, Royal College of Surgeons in Ireland, Busaiteen, BHR

**Keywords:** arab, body dysmorphia, body image, eating disorder, social media

## Abstract

Eating disorders are one of the most common psychiatric disorders. It has a significant impact on mortality that affects a huge percentage of the population. This study aims to understand the relationship and impact of the use of social media, such as Instagram (Meta Platforms, Inc., Menlo Park, CA), Snapchat (Snap Inc., Santa Monica, CA), Facebook (Meta Platforms, Inc., Menlo Park, CA), Twitter (X Corp., San Francisco, CA), TikTok (ByteDance Ltd., Beijing, China), and YouTube (Google LLC, Mountain View, CA), on body image and eating disorders in Arab countries. A literature review was conducted. A computerised search was performed on electronic websites such as PubMed, Scopus, and Google Scholar, limiting it to the past 20 years, focusing on participants with eating disorders and the use of social media. The results show that there is a strong relationship between social media and eating disorders, as social media has a negative effect on how people view their bodies and body image, which leads to an increase in the risk of eating disorders. The risk of eating disorders increases with the use of social media; therefore, preventative methods should be considered.

## Introduction and background

Each year, over 3.3 million lives are lost because of eating disorders (EDs) [1], with anorexia nervosa (AN) having the highest mortality rate of all psychiatric conditions [2]. One emerging risk factor linked to these disorders is the pervasive influence of social media, which has transformed how individuals perceive body image and beauty ideals [3]. While this relationship has been widely studied in Western societies [4], the cultural context of Arab countries remains underexplored [5]. 

EDs are serious mental health issues affecting people of all ages, genders, and cultures due to drastic changes in their eating practices, and they come in several different types, such as AN, bulimia, and binge ED. It also plays a significant role in the cause of morbidity and death among teenage girls and young adult women. AN is defined as dieting or not eating to a point where a person has a BMI less than 17.5 kg/m3 [6], while bulimia is described as a “chronic phase of AN” which is episodes of excessive food consumption followed by purging, laxatives or prolonged fasting and binge ED involves frequent episodes of overeating and experiencing loss of control over their eating behaviour [7,8].

Body dysmorphic disorder (BDD) consists of distress or preoccupation with perceived defects in physical appearance that are nonexistent or only slight. It is often associated with marked impairment in functioning, poor quality of life, and high rates of suicidality. The development of BDD has been linked to the passive use of social media and photo-editing apps [9].

Social media platforms, such as Instagram (Meta Platforms, Inc., Menlo Park, CA), TikTok (ByteDance Ltd., Beijing, China), and Snapchat (Snap Inc., Santa Monica, CA), promote highly curated, idealised images that can intensify body dissatisfaction, especially among adolescents and young adults [10]. It has been stated that from the beginning of the COVID-19 pandemic, media devices and internet access rapidly increased and have become part of our lives, as an Italian National Institute of Statistics (ISTAT) report found that over 72% accessed the internet via smartphones [11]. 

The Arab world presents a unique intersection of traditional beauty norms and rapid digital globalisation. The Arab world’s beauty-perception landscape exemplifies a cultural crossroads where inherited traditions meet the forces of digital globalisation. Rather than one displacing the other, both coexist and evolve together, creating a dynamic, hybrid beauty culture that reflects broader social transformations in gender, identity, and media. However, these deep-rooted traditions and modern global influences have the very real potential to clash, currently creating a precarious balance between Eastern and Western beauty norms. Beauty ideals in Arab societies have long been shaped by cultural, religious, and social values emphasising modesty, femininity, and natural elegance. These mores are bereft of any overt sexualisation. However, recently, the rise of digital globalisation, particularly through social media platforms, has introduced Western and East Asian beauty standards, global cosmetics brands, and influencer culture into Arab contexts. This convergence has generated a hybrid beauty culture that blends heritage and modernity in unprecedented ways. This review aims to discuss how social media impacts body image and EDs within Arab populations, accounting for culturally relevant risk and protective factors. The points raised may be potential guides for future interventions in this region [12]. 

Methodology

This is a review of the published literature. A search was conducted across multiple electronic databases, which included Scopus, PubMed, and Google Scholar, using relevant keywords: “social media,” “eating disorders,” “body image,” “body dysmorphia,” and “Arab countries.” The studies were limited to those published in the last 20 years (2005-2025), and both quantitative and qualitative studies were considered. The selection process followed the inclusion and exclusion criteria. The review only included articles that referred to Arab countries, were published in the English language, and were published 20 years ago. Articles that did not follow these criteria were excluded. 

This review used a narrative synthesis approach to summarise and interpret the findings of the included studies. Themes and trends related to social media usage patterns, specific platforms, content exposure, body image perceptions, disordered eating behaviours, and the prevalence of EDs were explored. Additionally, potential moderating factors, such as cultural norms, gender, and age, were examined to gain a comprehensive understanding of the contextual influences on the relationship between social media and body image-related outcomes in the Middle East.

## Review

Prevalence and impact of eating disorders and body image disorders on individuals

EDs, including AN, bulimia nervosa, and binge ED, affect individuals across all cultures and genders. These conditions are associated with significant psychiatric comorbidities, such as depression, anxiety, personality disorders, and substance abuse; they also carry elevated mortality risks [13], which increases the chance of death and mortality rate. EDs also have several medical complications, such as endocrine dysfunction, cardiovascular disease, and anaemia. AN has the highest fatality rate among psychiatric illnesses [14].

Given the three types of EDs, there is a need to assess the risk of developing any of the established types of EDs. A study done in Western settings has established that young women experience more EDs compared to men in early adulthood. This viewed relationship is accurate across all three distinct types of EDs. AN was eight times more prevalent in women compared to men. Women are at a higher risk of EDs than men, and this could be due to several factors that play a role in increasing the risk [15].

Social media has a profound impact on body image, which is the person’s perceptions, thoughts, and feelings about their body, and could later cause body image dissatisfaction or body dysmorphia [16], particularly among young women. As social media usage grows, so does the exposure to thin, muscular, and fit ideals, which can negatively affect how individuals perceive their bodies, fostering a specific beauty standard that others feel pressure to meet. Today, social media is the most prevalent form of communication, and the constant promotion of these idealised bodies intensifies the drive for thinness and body dissatisfaction among young females [17].

Although EDs and body image issues affect females, it is important to acknowledge that social media also impacts males in diverse ways. For men, the pressure is often to achieve a muscular and bulky physique, which can also lead to body image issues, body dysmorphia, and potentially EDs such as binge eating to bulk up [18].

In Arab populations, the prevalence of EDs is rising, particularly among young women, university students, and adolescents [19]. This increase is influenced by the unique cultural context of the Middle East, where Islam has a massive impact on daily life and regular ritual fasting is actively encouraged. As a result, reduced food intake may often be perceived as socially acceptable, masking ED symptoms under the guise of religious practices or dieting.

Cultural stigmas around mental illness often delay diagnosis, complicating the situation further [20]. Although little research has explored this relationship, a study that was conducted on both males and females engaging in intermittent fasting for 12 months and 30 days (about four and a half weeks) showed that there is a significant association with EDs. It can uniquely predict the risk of ED as it is identified as a major risk factor for recurrent binge eating and the development of bulimia [21].

Gender differences and psychological mechanisms at play

Gender differences play a significant role in shaping body image experiences [18]. Societal beauty ideals often emphasise thinness for women and muscularity for men, leading to distinct pressures and expectations. Women are frequently subjected to pressure to achieve a slender physique, often leading to heightened body dissatisfaction due to frequent comparisons with idealised images of thin models and celebrities portrayed in the media [22]. On the other hand, men often face pressure to achieve a muscular and lean physique, driven by the prevalence of athletic male bodies in the media. While EDs can affect individuals of any gender, women are more commonly diagnosed with conditions like AN and bulimia nervosa. In contrast, men are more prone to muscle dysmorphia, characterised by an obsession with attaining a highly muscular physique [23]. 

A study conducted among Arab adolescents found that female adolescents reported higher levels of body image dissatisfaction and disordered eating behaviours compared to males. This suggests that gender differences play a role in the impact of social media on body image and EDs in Arab countries, highlighting the need to consider these differences in the treatment and prevention of EDs globally, as females are at a higher risk of developing such disorders [24]. In the UAE, 29% of normal-weight women reported perceiving themselves as overweight, compared to only 8% of men. Females were more likely to diet, while males preferred exercise, suggesting gendered coping mechanisms influenced by both cultural and media pressures [25].

In addition to gender differences, psychological mechanisms play a crucial role in how social media affects body image. One such mechanism is social comparison theory, which suggests that individuals engage in upward social comparisons on social media, leading to lower self-esteem as they compare themselves to others perceived as having a more attractive or ideal body. This was assessed using the Sociocultural Attitudes Towards Appearance Questionnaire-4R (SATAQ-4R) [26]. This mechanism may be particularly relevant in Arab countries, where there is a strong emphasis on appearance and beauty standards.

Other moderating risk factors

A range of biological, psychological, and sociocultural factors contribute to the development of EDs. These include genetic predisposition, alterations in gastrointestinal microbiota, autoimmune responses, early life exposures during childhood and adolescence, specific personality traits (e.g., perfectionism or neuroticism), coexisting mental health disorders, socio-economic challenges, minority status, body image concerns, social pressures, and participation in elite-level sports. [27]. While the manifestations of these risk factors may vary between genders, research indicates that approximately 52% of males are at risk for developing EDs. Nonetheless, females remain at a disproportionately higher risk due to pervasive societal pressures to attain a thin and lean body ideal. This gender disparity reflects both differing cultural expectations and internalised beauty standards, which can significantly influence the onset and progression of disordered eating behaviours [28].

It has also been reported that experiences of abuse, trauma, and childhood obesity are strongly associated with the development of EDs. Co-occurring mental health conditions, such as personality and mood disorders, have been shown to exacerbate ED symptoms. Additionally, factors like higher educational achievement, body image concerns, and engagement with appearance-centric social media are linked to a heightened risk of ED symptoms, as they often foster insecurity and body dysmorphia in adults [26]. 

One of the most significant moderating factors influencing EDs was the COVID-19 pandemic. During the lockdown, binge eating increased among individuals in the general population due to the prolonged stay-at-home orders, which disrupted daily routines and created higher stress levels. Females were identified as being at a greater risk for pandemic-related binge eating. Those already suffering from symptoms of psychiatric disorders or stress due to the lockdown were at an even higher risk of using food as a form of comfort, leading to episodes of stress eating and binge eating. This pattern reflects the use of food as a coping mechanism during periods of emotional distress, which exacerbated disordered eating behaviours during the pandemic [24].

Moreover, mothers with a history of EDs pose a significant risk factor for their children’s body image and eating behaviours [29]. Studies have shown that children aged eight to 13 whose mothers had a history of EDs exhibited greater ED psychopathology related to EDs and engaged in more emotional eating compared to children of mothers with either emotional disorders or no psychiatric history. These children also demonstrated lower self-esteem and were more likely to internalise societal pressures to conform to slender ideals. Mothers with a history of EDs tended to have higher levels of weight concerns, which often manifest in more restrictive and intrusive feeding practices, leading to greater conflict around their children’s eating habits. Additionally, these mothers reported lower self-efficacy when it came to promoting positive body image and healthy self-esteem in their children’s development. The intergenerational transmission of disordered eating attitudes and behaviours underscores the profound influence that a material ED history can have on the body image and eating patterns of their children during the crucial development stages of childhood and early adolescence [30].

Protective factors and interventions

The term “protective factors” refers to elements that moderate the impact of risk factors on development and promote resilience. These factors are critical in mitigating the adverse effects of social media on body image and EDs, particularly in the Middle East. One potential protective factor is the practice of regular family meals, which is inversely associated with weight-related issues. A positive atmosphere during family meals can contribute to healthy eating habits. Research has also indicated that higher maternal BMI at age 10 is linked to reduced risk of AN and higher self-esteem in childhood. Furthermore, parental conversations about healthy eating are associated with the lowest prevalence of disordered eating behaviours [31].

In addition to family dynamics, positive body image campaigns and media literacy programs are vital in countering the harmful effects of social media. Social media can be leveraged to promote body positivity and diversity, with body-positive influencers and campaigns that highlight the beauty of all body types serving as a counterbalance to harmful and unrealistic societal ideals. To address the detrimental effects of social media on EDs and body dysmorphia, researchers and practitioners have proposed various preventive methods and interventions.

The treatment of EDs is highly intricate, often necessitating a multidisciplinary approach. International guidelines advocate for psychological therapies as the first-line treatment for individuals with AN. For adolescents with AN, manualized family-based therapy (FBT) is the preferred method. In adults with AN, recommended therapies include cognitive behavioural therapy for EDs (CBT-ED), focal psychodynamic psychotherapy (FPT), the Maudsley model of AN treatment for adults (MANTRA), or specialist supportive clinical management (SSCM). As for pharmacological treatments, antidepressants, particularly selective serotonin reuptake inhibitors (SSRIs), have been suggested as a supplementary option for individuals with binge-eating spectrum disorders. However, in the US, lisdexamfetamine is the only medication approved for binge-eating disorder (BED), and no medication has been approved for EDs in Europe, except fluoxetine for bulimia nervosa [32].

Results

Table 1 shows multiple studies that were conducted in different countries in the Middle East, including Saudi Arabia, Qatar, the United Arab Emirates, and Lebanon. It describes the relationship between social media use and how it affects body image and EDs. The first study conducted in Saudi Arabia with 1,010 participants showed that individuals with body dysmorphia were more likely to compare their appearance with that of famous people on social media. This suggests that the exposure to idealised and unrealistic body images on social media platforms may contribute to body dissatisfaction and the development of BDD, and further lead to EDs [33].

**Table 1:** An overview of some of the articles used in this review

Author (year)	Country	Sample/sex	SM measure	Eating disorder measure	Body image measure
Alsaidan et al. (2020) [33]	Saudi Arabia	1,010 participants	The use of SM	N/A	Body Dysmorphic Disorder Questionnaire
Qutteina et al. (2019) [34]	Qatar	1,418 participants	Popular social media platforms were assessed.	EAT-26	Not measured
Radwan et al. (2018) [35]	United Arab Emirates	662 participants (407 women and 255 men)	Media influences scale	EAT-26	Body Shape Questionnaire (BSQ-8)
Ateq et al. (2024) [36]	Saudi Arabia	1,483 Saudi adults	Social media usage	N/A	Body Dysmorphic Disorder Questionnaire
Berjaoui et al. (2023) [37]	Lebanon	1,048 Lebanese females	A questionnaire about social media use	N/A	BDD-Q and acceptance toward cosmetic surgeries (ACSS)
Alruwayshid et al. (2021) [38]	Saudi Arabia	204 students	Social media use	N/A	BSQ-8
Karam et al. (2023) [39]	Lebanon	292 university students	Bergen Social Media Addiction Scale (BSMAS)	N/A	Body Shape Questionnaire16-Item Version (BSQ-16)

In addition, a study that was conducted in Qatar with 1,418 participants revealed that intensive use of social media platforms had a positive association with increased disordered eating behaviours among young women. The study assessed multiple social media platforms, such as Instagram, Snapchat, Facebook, and Twitter. The findings show that there is a potential influence of these platforms in affecting and determining beauty standards and promoting an unhealthy relationship with food and body image. The study’s use of the 26-item Eating Attitudes Test (EAT-26) further confirmed the association between social media use and disordered eating behaviours [34].

Moreover, in the United Arab Emirates, a study involving 662 participants, including 407 women and 255 men, investigated the influence of the media on eating attitudes and body shape concerns among university students. The study utilised the media influence scale, the EAT-26, and the Body Shape Questionnaire (BSQ-8). The results showed that there is a high prevalence of EDs and body shape concerns in this population, suggesting that the media play a significant role in body image perceptions and influence disordered eating patterns among the students [35].

In Saudi Arabia, another study conducted with 1,483 Saudi adults examined the relationship between social media usage and BDD. The study revealed that 24.4% of the sample showed symptoms of BDD. In addition, participants who spent four to seven hours on social media had a 29% higher likelihood of experiencing BDD compared to those who used social media for less than an hour a day. This suggests that the duration of social media use has an effect and is a significant factor in the development of BDD [36].

In Lebanon, a study with 1,048 Lebanese females explored the prevalence of BDD and its association with social media use. The study used an online survey that included questions about social media use, sociodemographic characteristics, cosmetic interventions, and BDD. The results indicated that around 13.5% of the participants were positive for BDD. This highlights the potential negative impact of social media on body image perceptions and the development of BDD among Lebanese females [37].

Additionally, a study conducted with 204 students in Saudi Arabia showed the relationship between social media use and body dissatisfaction. The study used the BSQ-8 to measure body dissatisfaction levels. The findings revealed that social media was associated with increased levels of dissatisfaction among the students, indicating that exposure to idealised body images on social media leads to negative body image [38].

Finally, a study that was done in Lebanon, which contained 292 university students, examined the relationship between social media addiction, body image concerns, and various behaviours and feelings related to eating habits. The study used the Bergen Social Media Addiction Scale (BSMAS) to assess social media addiction, and they used the BSQ 16-item version (BSQ-16) to measure body image concerns. The results showed that social media addiction was strongly related to body image concerns and behaviours such as emotional overeating, food responsiveness, and feeling hungry [39].

In conclusion, these studies highlight the impact of social media on body image perception, eating behaviours, and the prevalence of BDD in various populations. There is a need for further research and the development of interventions to promote healthy body image and mitigate the adverse effects of social media on individuals’ well-being.

Limitations and strengths

This literature review offers several strengths, including a thorough exploration of the relationship between social media use and body image disturbances within diverse Arab cultural contexts. It emphasises how sociocultural norms, gender roles, and digital exposure interact to shape the risk of EDs. However, several limitations should be acknowledged. Research surrounding body image and EDs is currently well established. There are many validated diagnostic and screening tools employed to qualitatively and quantitatively assess BDD and EDs. In contrast, the methods for quantifying SM use are far less developed. Furthermore, a disproportionate focus on young women limits the generalizability of findings to other groups, including males, older adults, and non-urban populations.

Recommendations and future directions

To address the current research gaps, future studies should prioritise longitudinal designs to clarify the causal relationship between social media use and the development of EDs across diverse demographic groups, including males and older individuals. Expanding the scope of research to encompass a broader range of cultural contexts within the Arab world will offer a more nuanced understanding of how regional and societal differences influence body image and disordered eating patterns. Furthermore, intervention-based research, particularly programs centred on media literacy and culturally sensitive body positivity campaigns, is essential. Collaborative initiatives involving mental health professionals, educators, and social media platforms will be key in creating safer digital spaces and promoting healthier body image perceptions among users.

## Conclusions

Studies conducted in Arab countries, including Saudi Arabia, Qatar, the United Arab Emirates, and Lebanon, frequently report upon the significant impact of social media on body image dissatisfaction and BDD. Individuals with BDD are more prone to upward social comparisons, especially with celebrities and influencers, which fosters unrealistic beauty standards and heightened dissatisfaction with their physical appearance. Although these findings appear well established, critical gaps remain; most notably the underrepresentation of Egypt, one of the most populous Arab countries, and the limited focus on male populations, as most existing research centres on young females which underscores an urgent need for more comprehensive and inclusive research to address the mental health consequences of social media use and promote healthier body image perceptions across all demographic groups in the Arab world. These conclusions are drawn from a specified geographical area, and these may not be generalised to other regions of the globe. Moreover, this geographical area has a complex and distinct cultural character, which is noteworthy when discussing the specificity and generalizability of the various studies in the literature.

Traditional Arab beauty ideals often align with Islamic principles of modesty (ḥayā’). Arab beauty practices are rooted in notions of cleanliness, moral virtue, and social respectability, as opposed to manifestly sexualized imagery. However, there is evidence that younger Arab women are increasingly adopting global standards of beauty (e.g., contouring, cosmetic surgery) while reinterpreting them to fit local expectations of modesty and cultural identity. Platforms like Instagram, TikTok, and Snapchat have amplified the presence of global beauty trends in the Arab world. There appears to be a clear tension and negotiation between tradition and modernity. The coexistence of hijab fashion, modest beauty brands, and Western beauty influencers exemplifies a negotiation between local identity and global aesthetics. These cultural negotiations are allowing Arab women to redefine beauty beyond traditional constraints, using platforms to discuss self-expression, body positivity, and gender norms. These were often taboo topics for the older generations.
